# Patient empowerment: The need to consider it as a measurable patient-reported outcome for chronic conditions

**DOI:** 10.1186/1472-6963-12-157

**Published:** 2012-06-13

**Authors:** Marion McAllister, Graham Dunn, Katherine Payne, Linda Davies, Chris Todd

**Affiliations:** 1 Senior Lecturer in Genetic Counselling, Institute of Cancer & Genetics, Cardiff University, Heath Park, Cardiff, CF14 4XN, UK; 2 Health Sciences - Methodology, School of Community-Based Medicine, Manchester Academic Health Science Centre, The University of Manchester, Jean McFarlane Building (First Floor), Oxford Road, Manchester, M13 9PL, UK; 3 The School of Nursing, Midwifery and Social Work, Manchester Academic Health Science Centre, The University of Manchester, Jean McFarlane Building (Room 6.314b), Oxford Road, Manchester, M13 9PL, UK

**Keywords:** Patient empowerment, Healthcare evaluation, Patient reported outcome measures.

## Abstract

**Background:**

Health policy in the UK and elsewhere is prioritising patient empowerment and patient evaluations of healthcare. Patient reported outcome measures now take centre-stage in implementing strategies to increase patient empowerment. This article argues for consideration of patient empowerment itself as a directly measurable patient reported outcome for chronic conditions, highlights some issues in adopting this approach, and outlines a research agenda to enable healthcare evaluation on the basis of patient empowerment.

**Discussion:**

Patient empowerment is not a well-defined construct. A range of condition-specific and generic patient empowerment questionnaires have been developed; each captures a different construct *e.g.* personal control, self-efficacy/self-mastery, and each is informed by a different implicit or explicit theoretical framework. This makes it currently problematic to conduct comparative evaluations of healthcare services on the basis of patient empowerment. A case study (clinical genetics) is used to (1) illustrate that patient empowerment can be a valued healthcare outcome, even if patients do not obtain health status benefits, (2) provide a rationale for conducting work necessary to tighten up the patient empowerment construct (3) provide an exemplar to inform design of interventions to increase patient empowerment in chronic disease. Such initiatives could be evaluated on the basis of measurable changes in patient empowerment, if the construct were properly operationalised as a patient reported outcome measure. To facilitate this, research is needed to develop an appropriate and widely applicable generic theoretical framework of patient empowerment to inform (re)development of a generic measure. This research should include developing consensus between patients, clinicians and policymakers about the content and boundaries of the construct before operationalisation. This article also considers a number of issues for society and for healthcare providers raised by adopting the patient empowerment paradigm.

**Summary:**

Healthcare policy is driving the need to consider patient empowerment as a measurable patient outcome from healthcare services. Research is needed to (1) tighten up the construct (2) develop consensus about what is important to include (3) (re)develop a generic measure of patient empowerment for use in evaluating healthcare (4) understand if/how people make trade-offs between empowerment and gain in health status.

## Background

Healthcare evaluation relies heavily on measuring health outcomes. There have been recent calls for healthcare evaluation to take into account the perceived value of non-health outcomes such as empowerment, a psychosocial outcome [1]. The separation of health status from psychosocial outcomes was first advocated in chronic disease by Kleinmann in 1988 [2]. Since then, patient empowerment has gained credibility in healthcare, reflecting moves away from paternalist models towards more equitable/collaborative models of clinician-patient interaction, including shared decision-making [3,4].

### Defining patient empowerment

What is patient empowerment? There are many definitions, with most relating in some way to patients conceived as self-determining agents with some control over their own health and healthcare, rather than as passive recipients of healthcare [5-9]. Associated with this, are ideas of patient compliance and adherence. Compliance refers to the patient submitting to the authority of their healthcare provider, and arguably belongs within the paternalist paradigm; adherence refers to the patient voluntarily agreeing with a healthcare plan, perhaps developed through shared decision-making and belongs more within the empowerment paradigm [10]. Indeed, it has been argued that patient empowerment offers an alternative to compliance [5,6]. Most definitions of patient empowerment include some conceptualisation of personal control and self-efficacy/self-mastery [3-9]. The concept of empowerment could be viewed as another conceptualisation of the capability paradigm suggested by Sen and introduced into healthcare by Coast and colleagues [11-14]. Most patient empowerment definitions focus on individuals’ capacity to make decisions about their health (behaviour) and to have, or take control over aspects of their lives that relate to health. Assumptions are that empowered individuals will (a) make more rational healthcare decisions to maximise their health and wellness (b) decrease dependence on healthcare services and (c) ultimately contribute to more cost-effective use of healthcare resources. However, these consequences remain to be proven.

Some argue that empowerment may be context and population specific and that a universal definition may not be possible [15]. Culture, age and socio-economic resources undoubtedly influence empowerment, and the degree to which different social groups can be, and wish to be, empowered will differ. Others have argued that empowerment can be considered to be either a process or an outcome; that patients can be empowered by their healthcare providers through education, counselling, patient-centred care, and use of community coaches; and that patients can empower themselves through self-education, facilitated by the internet, or by participating in patient organisations or community activism [16].

### Operationalising patient empowerment

The focus of many patient empowerment initiatives has been to encourage patient self-management of chronic conditions, although it is not always clear whether empowerment is being considered as a process or as an outcome. In published trials of self-care interventions, outcomes such as self-reported health, healthcare service use and patient self-efficacy are measured [7,17,18]. However the ability of the outcome measures used to capture the patient benefits of these programmes has been questioned [18]. It is true that those outcomes together may represent an adequate operationalisation of self-management, as defined by Lorig and colleagues [8] to include medical or behavioural management, role management, and emotional management. That operationalisation of self-management assumes that changes in self-efficacy should correlate with changes in health status and clinical outcomes [8]. However, outcomes such as self-reported health, healthcare service use and patient self-efficacy fall short of an adequate operationalisation of broader definitions of patient empowerment as outcome, that include the physical, emotional, cognitive, and spiritual needs of people living with chronic disease [2-6]. So, some patient benefits, particularly those that belong in the psychosocial realm (emotional, cognitive, social and spiritual needs) may not be captured.

Despite the absence of a generally accepted definition of patient empowerment, questionnaires that aim to capture patient empowerment have been developed in some areas. However, many of the questionnaires developed capture limited definitions of patient empowerment. There are some generic and condition-specific validated questionnaires that could be classed as patient empowerment measures. Examples are shown in Table1, along with the constructs that are operationalised in those measures. All these questionnaires were developed independently using different (explicit or implicit) theoretical frameworks. It is clear that existing measures that capture (aspects of) patient empowerment are not comparable, and many capture limited conceptualisations of empowerment.

**Table 1:** Some examples of validated questionnaires capturing (aspects of) patient empowerment

**Measure**	**Conditions-specific or generic?**	**Construct operationalised**	**Reference**
Patient Enablement Instrument	Generic	Aspects of perceived control over illness	Howie et al. 1998 [19]
Patient Activation Measure	Generic	Activation levels (skills, knowledge, and beliefs needed by patients to self-manage, collaborate with healthcare providers and maintain their health)	Hibbard et al. 2005 [20]
The Empowerment Scale	Conditions-specific: Mental healthcare	Self-efficacy, power-powerlessness, community activism, righteous anger, and optimism-control over the future	Rogers et al. 1997 [21],
Diabetes Empowerment Scale	Conditions-specific: Diabetes	Self-efficacy	Anderson et al. 2000 [22]
Patient Empowerment Scale	Conditions-specific: Cancer	Use of coping resources, an aspect of personal control	Bulsara et al. 2006 [23]
Genetic Counselling Outcome Scale	Conditions-specific: Genetic conditions	Perceived personal control (cognitive, decisional and behavioural control), hope and emotional regulation	McAllister et al. 2011 [24]

### The policy landscape

In the UK, the Coalition Government has reiterated NHS commitment to patient empowerment with the publication in 2010 of the NHS White Paper: ‘Equity and excellence: Liberating the NHS’ [25]. The Health Minister Andrew Lansley said in his speech to the House of Commons “Patients will be at the heart of the new NHS. Our guiding principle will be ‘no decision about me, without me’. We will bring NHS resources and NHS decision-making as close to the patient as possible.” The 2010 White Paper takes forward strategies developed by Lord Darzi in his review to (1) give patients more choice and control over their healthcare and (2) make hospital funding contingent upon performance against a range of quality measures including Patient-Reported Outcome Measures (PROMs) [26]. Together, it is claimed that these initiatives will empower patients.

In the US, self-completion questionnaires are used to capture aspects of patient satisfaction, *e.g.* with the degree of involvement patients had with decision-making about their care. One example of this approach is the US Consumer Assessment of Healthcare Providers and Systems (CAHPS) suite of surveys and quality improvement tools. But approaches based on patient satisfaction do not capture true outcomes of a healthcare intervention. A true PROM would measure statistically significant change in a measurable outcome before and after an intervention, rather than simply asking about satisfaction afterwards. PROMs are measures, normally short self-completion questionnaires, used to capture aspects of patient health status or health-related quality of life (HRQL) that come directly from the patient [27]. A key feature of a PROM is that measurements do not involve attribution of patient states by a healthcare provider. PROMs are taking an increasingly important role in healthcare evaluation. PROMs development is also an important part of the US NIH roadmap, exemplified by the work of the Patient Reported Outcome Measurement Information Systems (PROMIS) initiative [28].

## Discussion

It is clear that health policy in the UK and elsewhere is prioritising patient empowerment and patient evaluations of healthcare. This article will argue for consideration of patient empowerment as a directly measurable patient-reported outcome (PRO) for chronic conditions. But first, we will consider some issues that arise in adopting the patient empowerment perspective.

### Arguments against patient empowerment

Are there difficulties in adopting the patient empowerment perspective? One counter-argument for patient empowerment is that the medical establishment wishes to push forward the empowerment agenda because if patients take ownership of their own healthcare decisions, then this enables clinicians to avoid responsibility [29]. However, this argument does not hold water in an age where the patient’s assessment of their healthcare is given prominence. Patients are unlikely to be satisfied by clinicians who shirk responsibility; they can tell the difference between genuine patient-centred care and avoidance of responsibility.

A further, perhaps a competing difficulty in adopting the patient empowerment perspective, is the potential impact that patient empowerment can have on clinician-patient relationships. Patient empowerment may require clinicians to relinquish some control (both informational and decision-making), and become less compliance-focused [5]. This would undoubtedly have some status implications, shifting the balance of power away from clinicians and towards patients, and this might be very uncomfortable for some - clinicians and patients alike. However, this shift in the balance of power is occurring in our society anyway, facilitated by the internet, by a service-oriented culture, and by the empowerment of patient organisations, who are increasingly setting the agenda in both healthcare and health research. Perhaps clinicians would do better to fully embrace this cultural change, by generating evidence that they can contribute to patient empowerment, rather than resisting it. Indeed, if clinicians can see evidence that their patients are benefiting by improved empowerment levels, this might help them to cope with their own feelings of powerlessness to improve patient health status in some circumstances.

A further counter-argument is that not all people want to be, or can be empowered at all times. For patients in acute care, there may be many who would prefer their doctor to make treatment decisions for them, at least in the short term. However, for patients with chronic conditions, relying on clinicians to be responsible for maintaining their health will become less tenable. Furthermore, if clinicians try to coerce people into making lifestyle changes to improve their (future) health, lifestyle changes that threaten fulfilment of their life goals, then this may lead to tensions in, and potentially, breakdown of the healthcare relationship. In practice, people with chronic conditions manage their own health over the long-term, with only occasional contact with health services.

### Patient empowerment as a directly measurable PRO for chronic conditions

Use of PROMs in healthcare evaluation is one way in which healthcare services are empowering patients, as this approach gives voice to the patients’ evaluations of the healthcare they have received, and links those evaluations to healthcare funding. Another approach could be to use PROM(s) capturing patient empowerment as outcome measures in their own right. This approach is aligned with (a) notions of shared decision-making that do not (necessarily) privilege health status as the only valued outcome [1] and (b) conceptualisation of patient empowerment, not as process, but as a useful, measurable health-related psychosocial outcome. Conceivably, such a construct could be measured using a PROM, enabling change over time to be captured. A recent WHO report called for better tools to measure empowerment [30]. A generic PROM of patient empowerment that could be used across a range of instances where patient empowerment is an explicit goal, such as in the liberated NHS (*e.g.* self-care/self-management programmes, personalised care plans, personal health budgets, shared decision-making) would enable evaluation of whether that goal has been achieved.

The George Institute have recently stated that (a) “Patient empowerment as an operational component of health reform must … have its value quantified” (p. 10) and (b) “what cannot be measured cannot be changed or even managed” (p. 11) [31]. We interpret this to mean that a valid and reliable measure of empowerment would be useful to evaluate healthcare interventions in a climate where patient empowerment is to be an integral part of healthcare reform. A PROM that measures change over time in empowerment levels would be useful to evaluate health and social care interventions, with appropriate case mix adjustment. This assertion is supported by qualitative research suggesting that providing patients with feelings of control (empowerment) over their health is highly valued and enables patients to better manage their lives, given the limitations imposed by their condition [32-35]. This approach has been explicitly implemented in some areas of healthcare such as diabetes [36-38] with some promising results.

Some consider patient empowerment to be an aspect of HRQL [39]. There is a plethora of generic and condition-specific measures of HRQL, but with general agreement that the best way to measure these constructs is by using PROMs. However measures of HRQL do not provide an adequate measure of patient empowerment because HRQL is itself an ill-defined construct. HRQL overlaps with health status and usually is understood to include one or more of the following dimensions: general health (physical and/or mental), functioning (physical, emotional, cognitive, sexual), social well-being, coping and adjustment, and existential issues [27]. Self-reported health status and psychosocial dimensions are poorly differentiated in HRQL. This makes it difficult to disentangle health status from psychosocial outcomes (emotional, cognitive, sexual, social well-being, coping and adjustment, spiritual) in intervention studies. This is important because if health status cannot be disentangled from psychosocial outcomes, then trade-offs cannot be explored between health status and psychosocial outcomes which in practice, patients may make. Enabling exploration of such trade-offs may facilitate a move away from compliance-orientation towards true patient empowerment, recognising that patients may also value outcomes other than health status, and potentially make it possible to demonstrate patient benefit even in situations where health status cannot be improved or maximised.

### A case study

Clinical genetics/genetic counselling services (CGS) provide a good case study for exploring psychosocial outcomes in chronic conditions because (a) genetic conditions are, by their very nature, chronic and life-long and (b) they can be studied in a health service context largely divorced from a focus on health status. This is because most genetic conditions cannot be cured, and currently for most genetic conditions, treatment is limited to symptom management or screening for complications. Although many CGS ‘patients’ are themselves healthy and come seeking a diagnosis in their child, or seeking information and counselling about their risk of developing or transmitting a genetic condition, the ‘chronicity’ of their ‘condition’ relates to the life-long and trans-generational impact of having a genetic condition in their family. In our work, exploring the best way(s) to evaluate a CGS, patient empowerment emerged from extensive qualitative research as a construct summarising the patient benefits of CGS [32,33]. The emergent model conceptualises empowerment as a multi-dimensional construct including

· cognitive control (sense-making - understanding the condition, why it happened, what help & support is available - “knowledge is power”)

· decisional control (having some options for managing the condition/risk and able to make informed decisions between options),

· behavioural control (able to do something to reduce harm or improve life for self/child(ren)/at risk relatives/descendents)

· emotional regulation (reflecting effective coping and adjustment)

· hope for the future (for self/relatives/future descendents).

This exploratory research identified patient empowerment as an important patient outcome, valued by patients and genetics clinicians.

Is there more that can be learned from the CGS case study? Genetic tests are rarely given routinely in the genetics clinic, unless a suspected diagnosis of a genetic condition can only be confirmed or excluded by a genetic test. Because of the eugenic past, modern genetic counselling endeavours to work within models emphasising non-directiveness and shared decision-making [40]. Goals of genetic counselling include facilitating informed patient decision-making and adjustment. Compliance is largely irrelevant. In this sense, CGS could be said to be ‘early adopters’ of the patient empowerment paradigm and that the rest of medicine is now beginning to catch up. The approaches and interventions of CGS could inform the design of interventions in other specialties aimed at increasing patient empowerment. Interventions offered by a CGS largely focus on information provision. This can be information about a genetic diagnosis, genetic risk information, information provided by a genetic test, or information about risk management options. But genetic counselling also includes facilitating decision-making about (1) how patients can best use genetic information given their personal life goals and values or (2) the appropriateness for the individual patient of having a predictive or prenatal genetic test - there may be far-reaching consequences for themselves and/or their (at risk) relatives. Patients are encouraged to reflect on multiple possible consequences for their own life circumstances before they make their decisions. Different people will make very different decisions, depending on their own life goals and values. The ‘intervention’ facilitates the ability to make decision(s); the same ‘intervention’ can result in very different actual decisions, or no decision yet, being reached by different patients. The important outcome for the patient is the ability to make those decisions.

### Implications for other chronic diseases

We believe that these approaches have implications for the design and evaluation of patient empowerment initiatives in other areas of healthcare *e.g.* self-management of chronic conditions such as depression, diabetes and asthma. Self-management programmes can be designed to empower people affected by chronic conditions to (1) identify, reflect upon and perhaps re-evaluate their life goals and values (2) understand how their behaviour influences their health and (3) develop the knowledge, skills, and confidence to make decisions about their health that best enable them to achieve their life goals and (4) stay committed when the ‘going gets tough’.

At present, we know that many patients with chronic conditions do not comply with what health care professionals consider optimal management strategies. However, we do not know whether this is mainly because patients want to comply, but struggle to and want help, or because they have priorities other than maximizing their health status. Previous research suggests that while some aspects of the decision-making of people with diabetes follow a narrow biomedical or strictly instrumental model of rational decision-making, that aims to maximize health status, other aspects do not [41]. Instrumental rationality focuses on outcomes and is concerned with the best means of achieving those outcomes. In the context of the biomedical model typically used for health, instrumentally rational choices will consider the set of all courses of action, a person’s beliefs about what the outcomes of each course of action is likely to be, and the person’s subjective valuation and ranking of those possible outcomes. This simple model underpins the use of measures of health status and quality adjusted life years (QALYs) in economic evaluations to inform health policy and health care decisions. This simple model can be expanded to incorporate important constraints. These are first, that people use a set of social norms to which they subscribe as part of the decision-making process. These shared rules are fundamental to a comprehensive model of rational choice. Secondly, individuals’ ability to find and use all relevant information to estimate the likely outcomes of all possible courses of actions is limited (which is necessary for instrumentally rational choices). As a consequence people treat the gathering and use of complex information as a cost, and will accept what appears to be a sub-optimal outcome to limit the search for further information. Finally, individuals use judgements rather than explicit calculations of expected costs and benefits in reaching decisions and making choices. These judgements incorporate their perceptions about what that choice signals to others as well as probabilistic, social, moral and ethical uncertainties about themselves and the world. These broader models of rationality provide insight into possible reasons for non-compliance with medically optimal strategies.

Combining this broader definition of rational decision-making with a patient empowerment approach would require clinicians to be more open minded and explicit about what outcomes patients might want, what norms and constraints the patient feels are important, the values and uncertainties the patient considers apply to themselves and the world. Within this broader framework, clinicians can explicitly work with patients to make informed decisions about what goals are important to them, and how best to reach those goals. Such an approach is likely to result in significantly greater positive participation in healthcare. Perhaps a PROM of patient empowerment, used alongside measures of self-reported health status and healthcare service use, could capture those other valued patient benefits even amongst those who do not derive dramatic health status benefits, or may even be used to offset losses in health status. This raises the question of which outcome we then ‘believe’ if there are gains in one and losses in the other. It is important to stress that the two types of outcome are not necessarily, or even usually, exclusive. Most healthcare professionals and patients strive to ensure that both outcomes are achieved. However, where trade-offs are made, it will be important to understand the trade off, or relative preferences that people have between health status and empowerment.

### Where do we go from here?

As yet there is no universally accepted generic model or measure of patient empowerment to enable comparative evaluations of different services. No two measures of patient empowerment have operationalised the same theoretical construct. Even so, a brief examination of some available measures confirms areas of convergence around dimensions of decision-making, control, self-efficacy (mastery), and self-management of disease (see Table1). So a case for developing a generic definition (theoretical framework) of patient empowerment can be made.

A robust generic theoretical model of patient empowerment would form a sound basis for selecting, developing or re-developing a generic PROM to capture patient empowerment for use in evaluating healthcare interventions and policies. But it is premature to choose or specify a generic model of patient empowerment at this point. More research is needed to develop an appropriate generic theoretical framework, such as a literature review to identify existing items/measurement domains, a theoretical review and classification of items and consensus development with stakeholders to identify what is important to include. This approach could develop a more “formal” or overarching generic construct of patient empowerment. This work should precede selecting, developing or re-developing a generic PROM to capture patient empowerment. Further work to explore relative preferences between health status and empowerment constructs, using a combination of qualitative research and discrete choice experiments, would also be useful.

Another challenge associated with introducing the measurement of empowerment relates to the current policy focus of maximising health status [1]. In the UK, the National Institute for Health and Clinical Excellence (NICE) has a clear rationale behind using health status and the QALY as the measure of patient benefit. This rationale is based on the premise that NICE is charged with providing evidence to inform how best (effectively and cost effectively) to use the finite ‘health’ care budget. The result is that the benefits of most technologies and interventions are valued by changes in health status and decisions to fund them are informed by evidence on the cost per additional QALY gained. The case study of CGS has indicated that not all healthcare interventions will show an improvement in QALYs. However, if we ‘solve’ this problem by using another measure, such as empowerment, we then create another problem. We are now valuing different healthcare interventions using different metrics, which means that another layer of value judgements must be made to answer: Which should we prioritise, improvement in health status or improvement in empowerment? It is possible to view healthcare interventions as being on a spectrum between those that purely maximise health (*e.g.* use of insulin to control diabetes) and those that purely maximise empowerment (eg. counselling interventions to improve ability to make informed health decisions in people living with diabetes). However, it is possible to conceive that the latter, if done well, could contribute significantly to the former, and indeed this is the basis of most self-management interventions. This issue indicates the need for more research, which should focus on understanding (a) how best to maximise both types of outcome for patients with chronic diseases and (b) any trade-offs that patients (current and future) make between maximising health and empowerment, using robust methodologies, such as discrete choice or best-worse experiments. Research of this kind could facilitate further moves away from compliance towards adherence and increasing patient empowerment, for example through patient-centred processes such as shared decision-making.

## Summary

In summary, patient empowerment is firmly on the health policy agenda in the UK and elsewhere. Patient evaluations of healthcare have become increasingly important, and strategies in place to increase patient empowerment in the NHS include wider use of PROMs, personal health budgets and personal health plans. There is also some evidence to suggest that patient empowerment may be a valued outcome from healthcare interventions that is related to, but independent of health status, and furthermore, that patients may value empowerment as an outcome even if they do not maximise their health status following a healthcare intervention. However, there is no widely accepted, effective method for evaluating whether healthcare strategies and interventions do, in fact, empower patients. Research is needed to clarify and tighten up the concept of patient empowerment and ensure patient, clinician and policy consensus on what is important to include. An appropriately robust theoretical framework of this kind would enable (re)-development of a robust generic measure of patient empowerment. The intention is not to replace existing healthcare evaluation methods, but to supplement them to facilitate re-orientation of services away from compliance towards increasing patient empowerment.

## Abbreviations

CAHPS, Consumer assessment of healthcare providers and systems; CGS, Clinical genetics/genetic counselling services; HRQL, Health related quality of life; NHS, National health service; PROM, Patient reported outcome measure; PROMIS, Patient-reported outcomes measurement information system; QALY, Quality adjusted life year; UK, United Kingdom; US, United States of America; WHO, World health organisation.

## Competing interests

(1) MM, GD and CT had support from MRC for the submitted work; (2) MM, GD and CT have no relationships with MRC that might have an interest in the submitted work in the previous 5 years; (3) their spouses, partners, or children have no financial relationships that may be relevant to the submitted work; (4) KP had support from RCUK for the submitted work; (5) KP has no relationships with RCUK that might have an interest in the submitted work in the previous 5 years; (6) Her spouse, partner, or children have no financial relationships with RCUK that may be relevant to the submitted work; (7) LD, GD and CT had support from HEFCE for the submitted work; (8) LD, GD and CT have no relationships with HEFCE that might have an interest in the submitted work in the previous 5 years; (9) their spouses, partners, or children have no financial relationships that may be relevant to the submitted work; and (10) MM, KP, LD, GD and CT have no non-financial interests that may be relevant to the submitted work.

## Authors' contributions

MM (a) conducted and led on publication of the research described in the case study, in collaboration with the other co-authors, (b) developed the arguments put forward in this article over the last 8 years, in consultation with each of the other co-authors, individually and collectively, (c) drafted the manuscript and co-ordinated input on the manuscript from the co-authors. GD & CT (a) contributed to design and analysis of the research described in the case study (b) contributed to developing the arguments put forward in this article over the last 4 years, in consultation with MM and the other co-authors and (c) reviewed and revised the manuscript critically for important intellectual content. KP & LD (a) contributed substantially to developing the line of argument and the interpretation of data over the last 7 years, and (b) reviewed and revised the manuscript critically for important intellectual content. All authors read and approved the final manuscript.

## Pre-publication history

The pre-publication history for this paper can be accessed here:

http://www.biomedcentral.com/1472-6963/12/157/prepub
